# Review of Chirped Fiber Bragg Grating (CFBG) Fiber-Optic Sensors and Their Applications

**DOI:** 10.3390/s18072147

**Published:** 2018-07-04

**Authors:** Daniele Tosi

**Affiliations:** 1 National Laboratory Astana, Laboratory of Biosensors and Bioinstruments, Astana 010000, Kazakhstan; daniele.tosi@nu.edu.kz; Tel.: +7-7172-70-5855; 2 Department of Electrical and Computer Engineering, Nazarbayev University, Astana 010000, Kazakhstan

**Keywords:** fiber optic sensors, Fiber Bragg Grating (FBG), Chirped Fiber Bragg Grating (CFBG), FBG sensors, photosensitivity

## Abstract

Fiber Bragg Gratings (FBGs) are one of the most popular technology within fiber-optic sensors, and they allow the measurement of mechanical, thermal, and physical parameters. In recent years, a strong emphasis has been placed on the fabrication and application of chirped FBGs (CFBGs), which are characterized by a non-uniform modulation of the refractive index within the core of an optical fiber. A CFBG behaves as a cascade of FBGs, each one reflecting a narrow spectrum that depends on temperature and/or strain. The key characteristic of CFBGs is that their reflection spectrum depends on the strain/temperature observed in each section of the grating; thus, they enable a short-length distributed sensing, whereas it is possible to detect spatially resolved variations of temperature or strain with resolution on the order of a millimeter over the grating length. Based on this premise, CFBGs have found important applications in healthcare, mechanical engineering, and shock waves analysis, among others. This work reviews the present and emerging trends in CFBG sensors, focusing on all aspects of the sensing element and outlining the application case scenarios for which CFBG sensors have been demonstrated.

## 1. Introduction

Within the broad field of fiber optic sensors [1], Fiber Bragg Grating (FBG) sensors are emerging as a prominent technology [2,3,4,5,6,7]. Since the demonstration of the photo-induced modulation of refractive index in the 90s [2], and the theoretical work that analyzes grating structures in fiber by means of coupled-mode theory [7] and layer peeling [8], FBG sensors have attracted significant interest thanks to their advantageous properties [6]: lightweight and compact form factor, immunity to electromagnetic fields, safety and biocompatibility, even in compliance to medical standards [9], fast response, suitability for harsh environments [9], and the possibility of creating sensing networks by means of time and wavelength division multiplexing [4].

Uniform FBGs are based on a periodical modulation of the refractive index in the core of an optical fiber [6,7], and they are the most popular grating-based type of technology. FBG sensors find current application in healthcare and medical devices [10,11], structural engineering [5,12], oil and gas [13,14], harsh environments [9], monitoring in nuclear plants [15], high temperature sensors [16]. Recent advances such as draw-tower fabrication [17] enable the inscription of FBG arrays.

From the electromagnetic point of view, the FBG is a periodic structure that resonates at one wavelength, namely the Bragg wavelength [2,3,4]. It reflects a very narrow spectrum around the Bragg wavelength, while it is transparent at the other wavelengths. An FBG can be considered as the optic equivalent to electrical band-reject filters, having an exceptional quality factor. The key characteristic of the FBG is that the Bragg wavelength changes linearly with strain and/or temperature, with typical sensitivity values of 1 pm/με (με being the microstrain units) and 10 pm/°C at 1550 nm [4]. Thus, the detection of the FBG spectrum with broadband interrogators and the estimation of the Bragg wavelength(s) [18] allow turning FBG(s) into sensing networks that directly detect temperature and/or strain [3,4], or can transduce other measurands such as pressure or curvature into strain [19,20].

The possibility to exploit photosensitivity to fabricate non-uniform gratings has enriched the typologies of FBG, which depend on the type of refractive index modulation and the fiber characteristics [6,7]. By acting on the refractive index amplitude, it is possible to generate apodized gratings [7,21], that have higher rejection of spectral side lobes, or phase shifted gratings [22]. A modern research trend exploits the possibility of tilting the grating profile, in order to excite cladding modes [23,24]; tilted FBGs have found application as refractive index sensors and biosensors [24]. FBGs inscribed in an etched fiber have also been used as biosensors thanks to their refractive index sensitivity [25]. FBGs inscribed in highly birefringent fibers can discriminate strain and temperature variations on the same grating [26].

All these types of grating are designed having a spatially uniform periodicity. By varying the period of the refractive index modulation along the grating length, it is possible to inscribe a chirped FBG (CFBG) [7,27,28,29,30,31,32,33,34,35,36,37,38,39]. In a CFBG, the Bragg wavelength varies along the grating, since each portion of the grating reflects a different spectrum [35]. The most important configuration is the linearly chirped FBG, whereas the Bragg wavelength varies linearly along the grating axis [35,36,37,38,39]. As a result, the CFBG has a reflection spectrum that is broader than uniform FBGs, and can range between few nanometers [31,32,33] to tens of nanometers [36,37,38] in full-width half-maximum bandwidth.

CFBGs became popular mainly for optical communications, as they enable dispersion compensation by means of introducing a differential group delay dependent on wavelength [27,28,29,30,31]. However, particularly in the last decade, CFBGs have gained substantial interest from the fiber-optic sensing community thanks to their key features and the inherent grating structure [33,34,35,36,37,38,39].

Most significantly, the key characteristic of the CFBG, in comparison with the uniform FBG, is the fact that the overall spectrum depends not only on the strain or temperature applied on the whole grating length, but rather on the temperature/strain recorded in each section of the grating [36]. In this framework, the use of a CFBG sensor can potentially detect rates of spatial changes of temperature or strain besides their temporal evolution [35]. In other words, CFBG can detect localized events, such as temperature hot spots [40] or strain discontinuities [41], besides detecting the values of such parameters.

While uniform FBG arrays have typical lengths up to 5 mm, and the minimum distance between each sensing element is usually limited to 10 mm [5,11], CFBGs have typical lengths of 15–50 mm [35,36,37,38] and potentially can discriminate spatial events with millimeter resolution [35]. From this perspective, CFBG sensors can be a valuable alternative to distributed sensors implemented with optical frequency domain reflectometry [42], optical backscatter reflectometry [43], or microwave photonics [44]. These short-length distributed sensors with narrow spatial resolution (1–10 mm in most devices [42,43,44,45]) allow the detection of physical parameters in every point of the optical fibers. The CFBG on the other hand can be described as a semi-distributed sensor [45]: it has an active length like a uniform FBG where the variation of strain or temperature is detectable, but within this region it can spatially resolve the temperature or strain profiles, like distributed sensors. CFBG sensors can use the same interrogation system as uniform FBGs [45], without requiring a complex and bulky interferometer.

These key characteristics define the applications for which CFBG sensors are attractive. Recent works have shown the application of CFBG sensors to healthcare [35], for mechanical crack detection [41], for heating localization [40], for detonation velocity measurement [34] among others. The common feature of these case scenarios is the possibility of using the CFBG not only as an individual sensor, but to detect the location of strain/temperature events along the grating length. Emerging works are also supported by the inscription of highly sensitive CFBG sensors on plastic fibers [39].

This work aims at providing a comprehensive review of CFBG sensors, highlighting the several applications of CFBGs in relation to each technological feature. In this review, only CFBGs having a significant chirp rate are considered. Section 2 describes the working principle of a CFBG, and describes a suitable model that allows determining how the CFBG spectrum changes as a function of different thermal or strain profiles. Section 3 describes the inscription methods for CFBG in different typologies of fibers. Section 4 describes interrogation and demodulation methods for CFBG, focusing on the methods based on spectral detection. Section 5 reviews the application of CFBGs, documenting the most recent works and the key features of the sensing element. Finally, Section 6 draws the conclusions.

## 2. CFBG Working Principle

**Figure 1:**
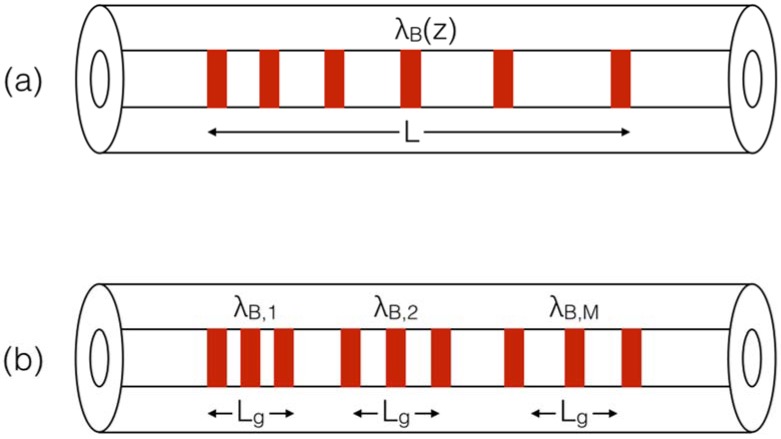
Schematic of a CFBG and its incoherent discretization method. (**a**) Sketch of a linearly chirped FBG; (**b**) correspondent discretization of the CFBG into M uniform FBGs.

The working principle of a CFBG extends from the uniform grating structure, and is sketched in Figure 1a. For a uniform FBG having no strain or temperature variations applied to it, the Bragg wavelength *λ_B_* is equal to [7]:

(1) $$\lambda_{B}=2\Lambda n_{eff}$$

where *Λ* is the period of the refractive index modulation, and *n_eff_* is the effective refractive index of the fiber core [4]. In a chirped FBG, periodicity of the modulation is not constant, but it changes along the propagation axis *z*; the function *Λ*(*z*) defines the chirp pattern. This implies that each different section of the grating reflects a different Bragg wavelength, and the overall spectrum of the FBG results from the spectrum of each section of the grating [35].

The method shown in [35,45,46,47,48,49] and outlined in Figure 1b provides a model of the FBG that can be defined as incoherent, as it assumes that the FBG can be modeled as a chain of *M* uniform FBGs each having different Bragg wavelength and with no intrinsic standing waves existing between each grating element. Calling *L* the grating length, and *M* the number of gratings, the length of each short grating element is *L_g_* = *L*/*M*. In [35,45] the grating has been discretized using *M* = 100–500. Within each grating, Erdogan’s coupled-mode theory (CMT) [7] can be used to estimate the reflectivity *R_i_*(*λ*) of each *i*-th layer as:

(2) $$R_{i}\left(\lambda \right)=\frac{\sinh^{2}\left(L_{g}\sqrt{k^{2}-\sigma_{i}^{2}}\right)}{\cosh^{2}\left(L_{g}\sqrt{k^{2}-\sigma_{i}^{2}}\right)-\frac{\sigma_{i}^{2}}{k^{2}}}$$

where *λ* is the wavelength, *kL_g_* is a unitless coefficient that defines the grating strength [6,7], and the term *σ_i_* contains the wavelength dependence for each layer. This is expressed, using CMT, as [7]:

(3) $$\sigma_{i}\left(\lambda \right)=\frac{\pi }{\lambda }\delta n_{eff}+2\pi n_{eff}\left(\frac{1}{\lambda }-\frac{1}{\lambda_{B,i}}\right)$$

where *δn_eff_* is the amplitude of the refractive index modulation. As in [35,49], the parameters of the refractive index modulation (*n_eff_*, *δn_eff_*, *k*) are assumed to be constant over the whole grating length and therefore they do not have a spatial dependency. The Bragg wavelength of each *i*-th layer, instead, is spatially varying. In a linearly chirped FBG [35,36,37,38,39,46,47,48,49] the reference value for the Bragg wavelength has a linear dependence upon the grating length:

(4) $$\lambda_{B}\left(z\right)=\lambda_{B}\left(0\right)+\xi z,\;\mathrm{for}\;0\leq z\leq L$$

which can be converted into the discretized grating model as:

(5) $$\lambda_{B,i}=\lambda_{B,1}+\xi \cdot iL_{g},\;\mathrm{for}\;i=1,2,\ldots ,M$$

In a linear CFBG defined in Equations (4) and (5), the chirp rate coefficient *ξ* is constant and defines the rate of spatial change of the Bragg wavelength within the grating structure. By inserting Equation (5) into Equations (2) and (3), it is possible to obtain the reflection spectrum *R_CFBG_* for each *i*-th grating composing the *M*-size structure; the overall spectrum can be obtained by multiplying the transmission spectra in cascade [35]:

(6) $$R_{CFBG}\left(\lambda \right)=1-\prod_{i=1}^{M}\left[1-R_{i}\left(\lambda \right)\right]$$

The operation in Equation (6) underlines the absence of coherency between each layer composing the grating. This assumption is compatible with most FBG interrogators based on a white-light setup as shown in [18,35,45], whereas the interrogation is based on a broadband source such as an infrared light-emitting diode (LED), a superluminescent LED [45], or an amplified spontaneous emission (ASE) source, and a spectrometer for detection. This working principle is implemented in commercial systems such as [50,51,52].

From the sensing perspective, the CFBG behaves as a cascade of FBGs, whereas each Bragg wavelength shifts as the grating is exposed to a temperature or strain variation [4]. As shown in [35,49], each *i*-th Bragg wavelength is sensitive to the strain variation Δ*ε_i_* and/or to the temperature variation Δ*T_i_* observed on the *i*-th element of the CFBG. For small values of strain and temperature [6], a linear sensitivity is observed:

(7) $$\lambda_{B,i}=\lambda_{B,i,ref}+s_{T}\Delta T_{i}+s_{\varepsilon }\Delta \varepsilon_{i}$$

The *i*-th reference wavelength *λ_B,i,ref_* is observed in reference conditions in absence of temperature or strain variations. The sensitivity terms refer to the change of Bragg wavelength for the CFBG, and are the same terms that are observed for a uniform FBG [4,5,6,7]; for an FBG operating in the infrared around 1550 nm, typical coefficients are *s_T_* ≅ 10 pm/°C for thermal sensitivity, and *s_ε_* ≅ 1 pm/με for strain sensitivity.

The key characteristic of the CFBG is the capability of working not only with spatially constant temperature/strain values, but to interpret profiles of strain or temperature, respectively Δ*ε*(*z*) and Δ*T*(*z*), which depend on the application where the CFBG is used for. For example, in [35] the CFBG is used in a thermal ablation application where the temperature profile has a Gaussian shape:

(8) $$\Delta T\left(z\right)=T_{0}\cdot e^{-\frac{{\left(z-z_{0}\right)}^{2}}{2v^{2}}}$$

which depends on the three parameters of a Gaussian function (*T*_0_, *z*_0_, *v*); in [53] an inclinometer has been documented having a strain linear profile that can be approximated as:

(9) $$\Delta \varepsilon \left(z\right)=\varepsilon_{0}+\varepsilon_{1}z$$

**Figure 2:**
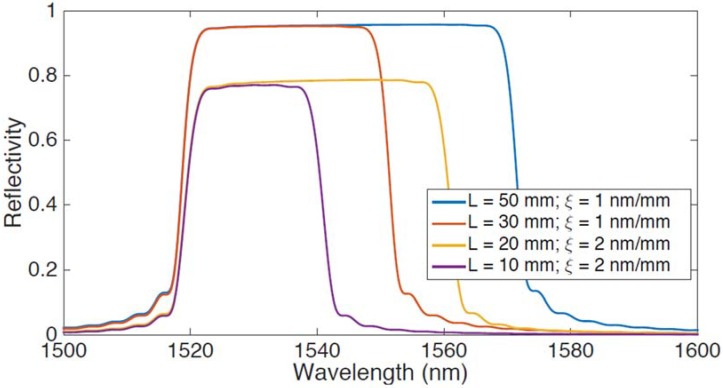
Simulation of CFBG spectra using the CMT-based model. The chart shows spectra having different length *L* ranging between 20 mm and 50 mm, and chirp rate coefficient *ξ* equal to 1–2 nm/mm; the other grating parameters are *δn_eff_* = 10^−6^, *n_eff_* = 1.5, *λ_B_*(0) = 1520 nm, *kL_g_* = 0.4 and the discretization step is *L_g_* = 0.2 mm.

The CMT-based FBG-cascade model for the CFBG is shown in Figure 2, where the reflection spectrum of a CFBG is shown for different grating profiles. The chart shows the spectrum simulated for different CFBG, all having different length *L* and chirp rate *ξ*, the same discretization step *L_g_* = 0.2 mm, and the same grating parameters (*kL_g_* = 0.4, *n_eff_* = 1.5, *δn_eff_* = 10^−6^, *λ_B_*(0) = 1520 nm). It can be shown that the CFBG appears as a flat spectrum, covering a bandwidth equal to the product *L* × *ξ*, and having a reflectivity that depends on the chirp rate. The CFBG used in applications such as [36,38,49,53] have bandwidth that can exceed 10 nm, similar to the spectra in Figure 2; in other applications where the chirp rate is limited by the FBG inscription setup [39], the CFBG has a narrower bandwidth limited to few nanometers.

**Figure 3:**
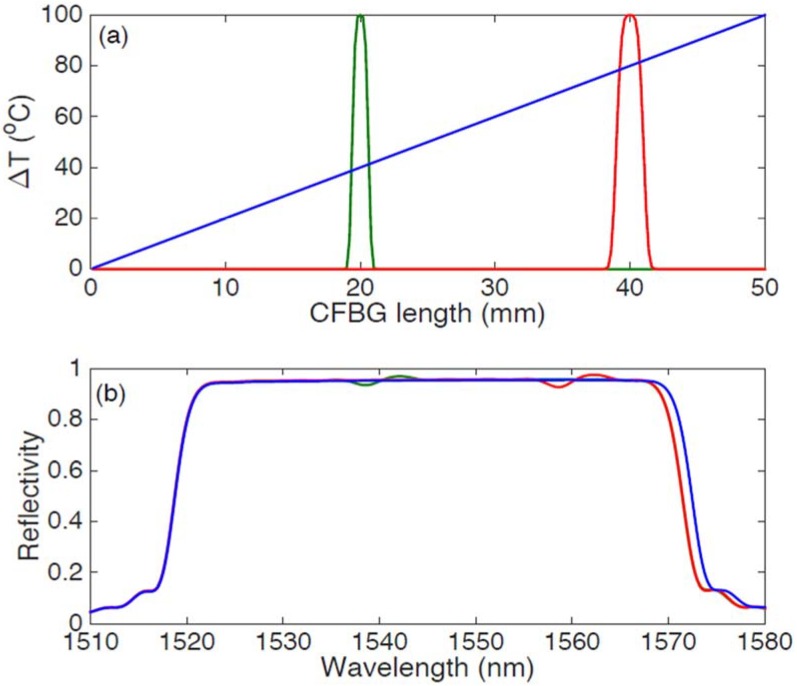
Simulation of the variations of CFBG spectra with CMT model, exposed to different temperature pattern. (**a**) Temperature variations applied to a 50-mm long CFBG with 1 nm/mm chirp rate; (**b**) Obtained CFBG reflection spectrum for each temperature profile. Each profile is displayed with the same color in the two charts.

In Figure 3 the concept of temperature profiling through CFBG sensors is exposed. Three different profiles are simulated in Figure 3a, including a linear profile throughout the whole CFBG length, that can mimic the temperature obtained in a gradient setup [35], and two Gaussian profiles with peaks in a different part of the grating length that mimic a thermo-therapies temperature pattern [38,45,46,47,48,49]. The grating parameters are the same of Figure 2, including 50 mm length and 1 nm/mm chirp rate, and the temperature coefficient has been set to 10 pm/°C. The resulting spectrum is shown in Figure 3b: for a linear profile, we observe a change of the grating bandwidth [29,31], that in this case enlarges due to a positive slope. For a Gaussian-shaped temperature pattern that is experienced in the inner part of the grating, the spectrum maintains its original shape on the edges, and a fluctuation on the spectrum is observed in the inner part of the bandwidth in correspondence to the location of the peak. The CMT based simulations show that the spectrum of the CFBG responds in different ways to different temperature or strain profiles: the chirp rate emphasizes this dependence on a broad spectral region.

The CMT-based model of Figure 3 assumes that no standing waves exist between each section of the CFBG. In order to adjust the model to a laser-based interrogator [18,54], it is possible to adjust the model by calculating the transmission matrix of each layer of the grating. This model has been implemented by Skaar’s layer peeling technique [8,55] and in the recent work of Palumbo et al. [36].

From the point of view of CFBG spectra, by comparing the filter cascade and the transmission matrix models, the main parameters such as full-width half-maximum (FWHM) bandwidth, reflectivity, and spectral response to temperature or strain patterns are similar; the main difference is the presence of minor spectral ripples within the CFBG bandwidth that can be observed with coherent sources [36].

## 3. Inscription of CFBG Sensors

The majority of CFBG devices used for sensors are inscribed using the phase mask technique [2,6,56], that makes use of a diffractive element and a mid-power UV, KrF, or Ti:sapphire laser to create a refractive index modulation pattern; these standards CFBGs have been used in many applications [35,36,38,40,45,57]. An emerging trend in fiber optic sensing is the inscription of CFBGs in non-standard fibers or using specialty phase masks [37,58,59,60,61,62,63]. The methods and reports on CFBG inscribed for sensing applications are listed in Table 1, and discussed in this section.

### 3.1. Phase Mask Inscription

**Figure 4:**
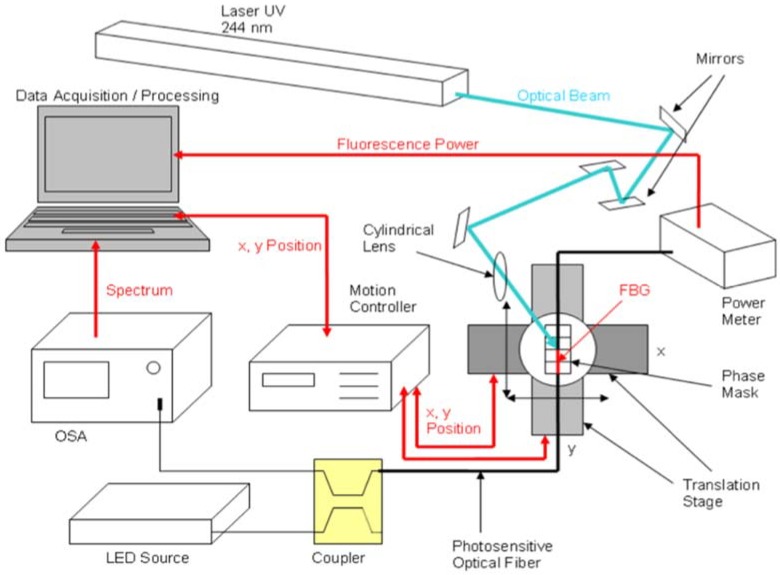
Schematic of phase mask inscription setup.

**Figure 5:**
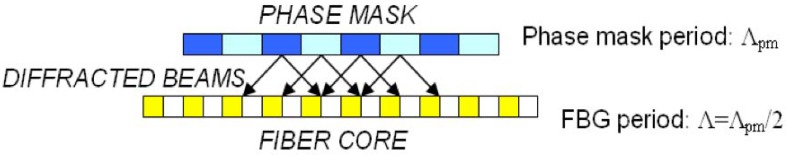
Schematic of the phase mask diffraction principle.

A setup for inscription of Bragg gratings based on a phase mask is shown in Figure 4, sketching the process used in [64]. The phase mask inscription exploits the photosensitivity of optical fibers [56], which determines the change of refractive index of the optical fiber core when exposed to an intense UV or KrF light. The phase mask is a diffractive element, usually fabricated with photolithography [2,6], that implements the refractive index modulation as sketched in Figure 5. When exposed to UV light, the phase mask cancels the 0-th order of diffraction, while having large diffractive energy for the ±1st orders of diffraction.

The resulting effect is that a period of refractive index modulation is induced in the fiber by means of constructive and destructive interference of the beams diffracted by the phase mask [4]. For a phase mask with pitch *Λ_pm_*, the resulting period of the FBG refractive index modulation is *Λ_pm_*/2 [6]. In order to fabricate a chirped FBG, it is necessary to have a chirped phase mask in which the period linearly varies along the fiber axis *z* [65,66], in order to obtain a linear variation of the Bragg wavelength corresponding to the chirp rate.

The setup for inscription allows scanning the whole grating length longitudinally. The setup in Figure 4 is based on a 244-nm UV laser, while other setups can use an Ar-ion laser [40], an excimer laser [63], or a KrF laser. After an optical path based on a beam expander and a collimator, the laser is focused on the phase mask, mounted on a *xy*-translation stage in order to scan all the grating length. The phase mask is mounted on top of the stripped fiber that hosts the FBG. The motion controller is set to scan the grating length longitudinally and compensating for eventual misalignments; this can be checked by monitoring the fluorescence power emitted from the fiber with a power meter [6]. During the inscription, the spectrum of the FBG is monitored with a white light setup, that in Figure 4 is an amplified spontaneous emission (ASE) connected to an optical spectrum analyzer (OSA).

The phase mask setup shifts the complexity of CFBG inscription to the phase mask, that is the element that contains the refractive index modulation. The process of inscription of FBGs through a phase mask has been consolidated in recent years, and allows inscribing gratings with FWHM of tens of nanometers and chirp rate typically ranging between 0.5 nm/mm to 2 nm/mm. From the industrial point of view, the process has been industrialized and consolidated [67]. One of the most recent developments in CFBG inscription machines is the NORIA instrument from Northlab Photonics [68], that embeds the whole FBG inscription setup and spectral monitoring in a single machine. The setup is equipped with phase mask mounted on a rolling wheel that allows the inscription of chirped FBGs with several length and chirp rates. In modern setups, the photosensitivity of standard single-mode fibers (SMF) is sufficient to inscribe a CFBG with high reflectivity; in addition, it is possible to use H_2_-loading [69] to increase the photosensitive effect.

CFBGs inscribed on single-mode fibers have been used in several applications. Among others, Korganbayev et al. [35] demonstrated a temperature sensor with a CFBG on SMF having 50 mm length and 0.8 nm/mm chirp rate, as well as 15 mm length 1.33 nm/mm chirp rate; a similar design has been chosen by Palumbo et al. [36] (45 mm length, 1.24 nm/mm) and Bettini et al. [57] (30 mm, 1.5 nm/mm). Nand et al. [40] inscribed a standard linear CFBG on H_2_-loaded fiber with an Ar-ion laser, having 25 mm length and 1.89 nm/mm chirp rate. All the mentioned gratings operate in the near infrared, within the third optical window centered at 1550 nm.

### 3.2. CFBG on PMMA Fibers

The main rationale for using polymer optical fibers (POFs) based on polymethyl methacrylate (PMMA) compound, in lieu of standard glass fibers for the inscription of CFBG sensors, is the change of thermal sensitivity [70]. While glass fibers achieve typical thermal sensitivity values of 10–15 pm/°C and strain sensitivity around 1 pm/με at 1550 nm, POF fibers have a larger thermal response, usually having negative sign (i.e., the larger the temperature, the smaller the Bragg wavelength).

**Figure 6:**
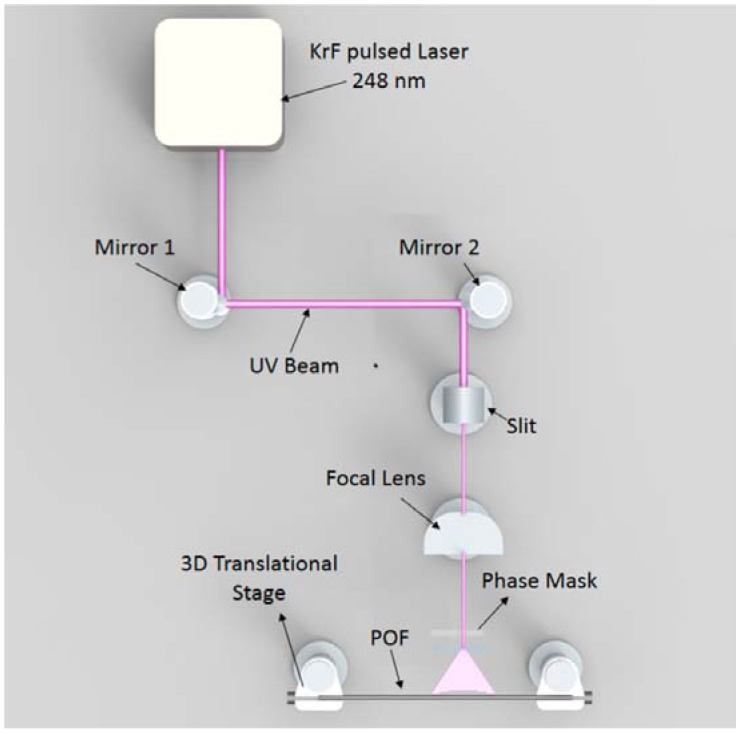
Schematic of phase mask inscription setup based on KrF pulsed laser for inscription of gratings on PMMA fibers, reported by Marques et al.; image from [70].

Among the several setups designed for POF FBGs, the KrF-based setup described by Marques et al. [70] allows a high efficiency and a short inscription time; the setup is sketched in Figure 6 (image from [70]). The laser is a KrF excimer laser at 248 nm emission wavelength, with 15 ns pulse duration. The system routes the pulsed light through an air path, and focalizes on a POF fibers mounted on a 3-dimensional translational stage, with the phase mask laying on the exposed fiber. The reported results shown an inscription time of 14–25 s depending on grating length and fiber compound.

The two most significant results related to the inscription of CFBGs on POF fibers are reported in [37,58]. In 2017, Marques et al. [58] reported a linearly chirped FBG inscribed on a step-index PMMA fiber having 8/210 μm core/cladding diameter, and both core and cladding in pure PMMA without dopant. The grating has length of 25 mm and 3.9 nm bandwidth, resulting in a chirp rate of 0.16 nm/mm. The thermal and strain sensitivity values recorded are −131.1 pm/°C and +1.77 pm/με, resulting in a ~10× magnification of the temperature sensitivity. In 2018, Min et al. [37] reported a CFBG on a photosensitive POF fiber. The fiber core is doped with BDK (benzyl dimethyl ketal), which increases the photosensitivity and allows the fabrication of a CFBG with a single laser pulse. The CFBG has been fabricated on a tapered POF fiber, which results in a change of FWHM bandwidth when the grating is subjected to strain. In [37], the authors observed a change of FWHM between 0.2 and 1.2 nm for a grating with 10 mm length. Sensitivity values are lower than [58] and have been estimated as −56.7 pm/°C and +0.71 pm/με.

Overall, the CFBG inscription on PMMA fibers is an emerging topic and the higher sensitivity to temperature is an important feature for magnifying the variations of optical spectra when the CFBG is subjected to a temperature variation. A significant step towards PMMA-CFBG sensors would be the increase of chirp rate coefficient, to date about one order of magnitude lower than in standard CFBG inscribed on telecom fibers.

### 3.3. Wideband CFBG

Bernier et al. [61] reported in 2009 a method for inscription of CFBGs having FWHM bandwidth significantly larger than conventional gratings. The inscription method is based on a femtosecond Ti:sapphire laser (35 fs) with a 1 kHz repetition rate, and a phase mask with a large chirp rate. The authors presented 3 results: (1) a CFBG fabricated on a standard SMF fiber having 25 mm length and 85 mm FWHM (3.4 nm/mm); (2) a CFBG fabricated on a H_2_-loaded fiber having 25 mm length and 206 nm FWHM (8.24 nm/mm), covering the S + C + L bandwidths; (3) a CFBG fabricated on a H_2_-loaded fiber having 35 mm length and 310 nm FWHM (8.85 nm/mm), covering the E + S + C + L + U bandwidths.

The increased bandwidth and chirp rate coefficient reported in [61] are significant with respect to standard CFBGs operating around 1 nm/mm, as the results are in excess of 8 nm/mm. The application of gratings with such high FWHM is hampered by the availability of components for the interrogation systems: as a benchmark, common superluminescent LED (SLED) [71] and spectrometers [52] operating in the near infrared achieve a usable bandwidth up to 80 nm, while broadband interrogators based on scanning laser and InGaAs photodetector [54] achieve a 160 nm usable bandwidth. The use of grating having such >200 nm bandwidth is limited to a system that has a supercontinuum source and a detector based on an optical spectrum analyzer (OSA). Both instruments are available either as benchtop or as original equipment manufacture (OEM) devices for integration, but are bulky and more expensive than standard interrogators. In addition, the use of an OSA for detection of a large bandwidth results in a slow measurement (several seconds of response time) as opposite to 1–10 Hz of static interrogators and 1 kHz of dynamic interrogators (typical values).

### 3.4. Draw-Tower CFBG

The possibility of inscribing FBGs with a draw-tower method [17,51] instead of a single phase mask has significant implications on the practical arrangement of the sensing networks. The most important factor is that, by inscribing the FBG during fiber drawing, it is possible to maintain the protective jacket of the optical fiber. FBGs inscribed by phase mask instead require stripping the fiber, a procedure that weakens the tensile strength of the fiber; it is possible to recoat the fiber with a polymer buffer, but this process increases the fiber thickness, may alter the sensitivity, and it is still less resistant than the original fiber.

The work of Idrisov et al. [59] attempts at the inscription of a linearly chirped FBG into a glass optical fiber, and it can significantly extend the process industrialized in [17,51] to chirped grating profiles. The technique reported in [59] implements a step-chirped FBG, which is realized as a cascade of narrow uniform FBGs each having a different index modulation pitch. The result is a spectrum characterized by significant ripples and having 0.5 nm FWHM on a 5 mm grating length, thus achieving a low chirp rate (0.1 nm/mm).

### 3.5. Tunable Phase Mask CFBG

The work reported by Voigtlander et al. [62] in 2009 proposes a different concept for the implementation of a phase mask technique. Instead of fabricating a silica phase mask with photolithography [2,6], and encode the refractive index modulation on the phase mask itself, in [62] the authors use a phase mask with a high thermal expansion coefficient, that can be stretched during the FBG inscription.

In the proposed setup, the phase mask is made of PMMA, having a high thermal expansion coefficient that results in a change of pitch length when the phase mask is heated. The mask is mounted on a set of Peltier cells, mounted aside of the scanning direction and controlled in real time during the inscription, that is based on a Ti-sapphire amplified laser. By tuning the phase mask acting on the Peltier cells, the authors have demonstrated a chirped FBG with FWHM up to 2 nm (chirp rate up to 0.1 nm/mm). Although the chirp rate is not large, this method allows a real-time control of grating properties, such as the chirp rate.

### 3.6. Regenerated CFBG

Qiao et al. [63] reported a thermally regenerated CFBG in a standard SMF fiber, which is resistant to high temperatures. The process of annealing has been used in [63] in order to form gratings that are stable at high temperatures, starting from a seed grating. The process starts from a 20-mm CFBG fabricated on a H_2_-loaded SMF—28 fiber using a KrF excimer laser. The initial CFBG is then regenerated by means of several thermal cycles reaching ~800 °C temperature, and allows the grating to sustain high temperature from 25 °C to 1000 °C. The FWHM of the CFBG appears to reduce during annealing, to a final value of 26.3 nm (1.31 nm/mm), and the final sensitivity observed is 15.1 pm/°C.

### 3.7. CFBG in Microfiber

Xiao et al. [60] reported a CFBG (5.5 nm FWHM) inscribed in a microfiber, that has a progressively reducing fiber diameter. The process of reducing the fiber cladding of an FBG has been well documented in [25] and it results in exposing the FBG to the external refractive index. Optically, the principle of operation is a change of the effective refractive index as a function of the external refractive index; it is due to the different index contrast between cladding and outer medium, and it results in a change of Bragg wavelength. This process is exploited in [60], using a CFBG in lieu of a uniform FBG; the authors documented a chirp rate of 1.57 nm/mm and a sensitivity of −5.2 nm/RIU (refractive index units).

## 4. CFBG Interrogation

### 4.1. CFBG Interrogation

**Figure 7:**
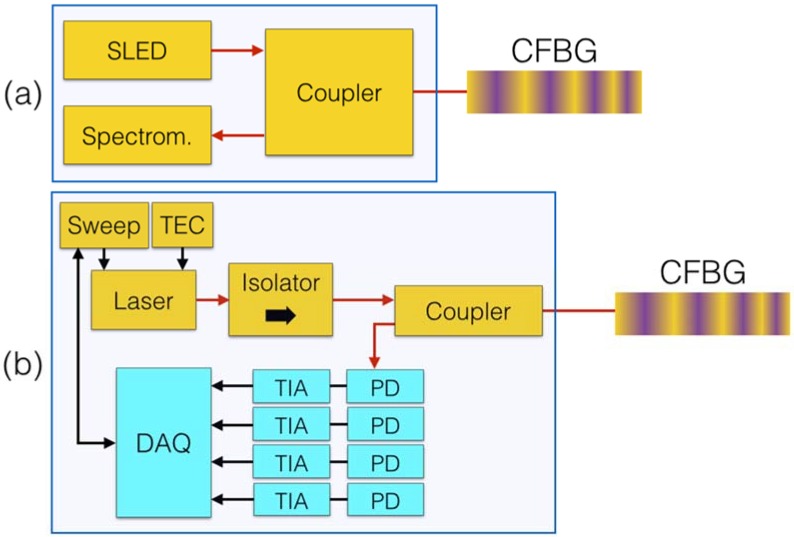
Schematic of CFBG interrogators, sketched as single-channel systems. (**a**) White light setup based on a spectrometer; (**b**) scanning laser based setup.

The interrogation of CFBG sensors is performed by detecting the spectrum of the Bragg grating within its window of operation, which allows further processing the results and extracting the temperature pattern. Two systems have been consolidated, based on two principles [18,45] as sketched in Figure 7: white light setup and scanning laser system.

In a white light setup sketched in Figure 7a, a broadband optical source, such as a fiber-coupled LED, a superluminescent LED [71], or an ASE is used as a light source, while the detector is a spectrometer [52] operating on the same bandwidth of the optical source. By means of a fiber coupler or circulator, it is possible to route light to the CFBG sensor and collect the reflected spectrum on the spectrometer. This method is fast and has no scanning part, but the resolution is limited by the spectrometer to typical values of 78–156 pm [18,52] on the window of operation, thus limiting the capability of detecting small spectral changes.

The scanning laser system, sketched in Figure 7b, aims at detecting the CFBG spectrum point-by-point [54], decomposing the components reflected at each wavelength. The source is a fiber-coupled scanning laser, controlled by a sweep function generator and stabilized by means of a thermo-electric controller (TEC). The detector is a photodiode (PD), usually having small active area and InGaAs semiconductor material, subsequently amplified by a transimpedance amplifier (TIA). A high-speed data acquisition (DAQ) hardware maintains the synchronization between the sweep function generator and the acquired spectral data. Systems such as [54] can achieve a resolution of 8 pm, over 20,000 wavelength values; however, the detected spectra are more noisy, and the laser scanning function requires a precise stabilization.

### 4.2. CFBG Parameters Estimation

Several methods have been proposed for the CFBG demodulation, in order to convert the CFBG spectrum detected by a method in Figure 7 into the variation of temperature or strain. The work of Saccomandi et al. [38] proposes the analysis of the two main CFBG parameters: the central wavelength *λ_C_* and the FWHM.

The central wavelength can be calculated with the methods reviewed in [18]. The method used for the computation of the central wavelength is based on a centroid method. Assuming that the reflection spectrum of the CFBG *R_CFBG_*(*λ*) is sampled on the wavelengths *λ*_1_, *λ*_2_, …, *λ_K_*, the central wavelength can be calculated using the centroid algorithm:

(10) $$\lambda_{C}=\frac{\sum_{i=1}^{k}R_{CFBG}\left(\lambda_{i}\right)\cdot \lambda_{i}}{\sum_{i=1}^{k}R_{CFBG}\left(\lambda_{i}\right)}$$

The FWHM can be estimated by implementing the following steps [18]:

(11) $$R_{th}=\max\left(R_{CFBG}\right)$$

(12) $$\lambda_{th}=\lambda_{R_{CFBG}\left(\lambda \right)>R_{th}}$$

(13) $$FWHM=\max\left(\lambda_{th}\right)-\min\left(\lambda_{th}\right)$$

which consists of setting a threshold at half of the maximum reflectivity, and evaluating the whole range of wavelength having a CFBG reflectivity higher than the threshold.

The work in [38] shows that the knowledge of the central wavelength allows measuring the average temperature of the CFBG, while the enlargement of the FWHM corresponds to the increase of thermal gradient recorded within the CFBG.

### 4.3. CFBG Spectral Reconstruction

As previously anticipated, a key feature of the CFBG sensors, that differentiates this class from uniform FBGs, is the fact that the spectral changes recorded by the CFBG are directly tied to the temperature/strain profiles that cause these changes. If the temperature or strain pattern along the direction *z* exhibits a pattern that is known a priori, or that can be assumed to be a function depending on some parameters (for example a polynomial of specific order, or a Gaussian function that depends on amplitude, variance, and central value), it is possible to run an optimization technique that compares the measured spectrum with the CFBG spectrum subjected to the temperature or strain pattern, until the best match is found. This approach, here labeled spectral reconstruction (because it aims at reconstructing the spectrum of the measured CFBG from its reference value and temperature or strain sensitivity), has been successfully demonstrated in [35,45,46,47,48,49,57].

**Figure 8:**
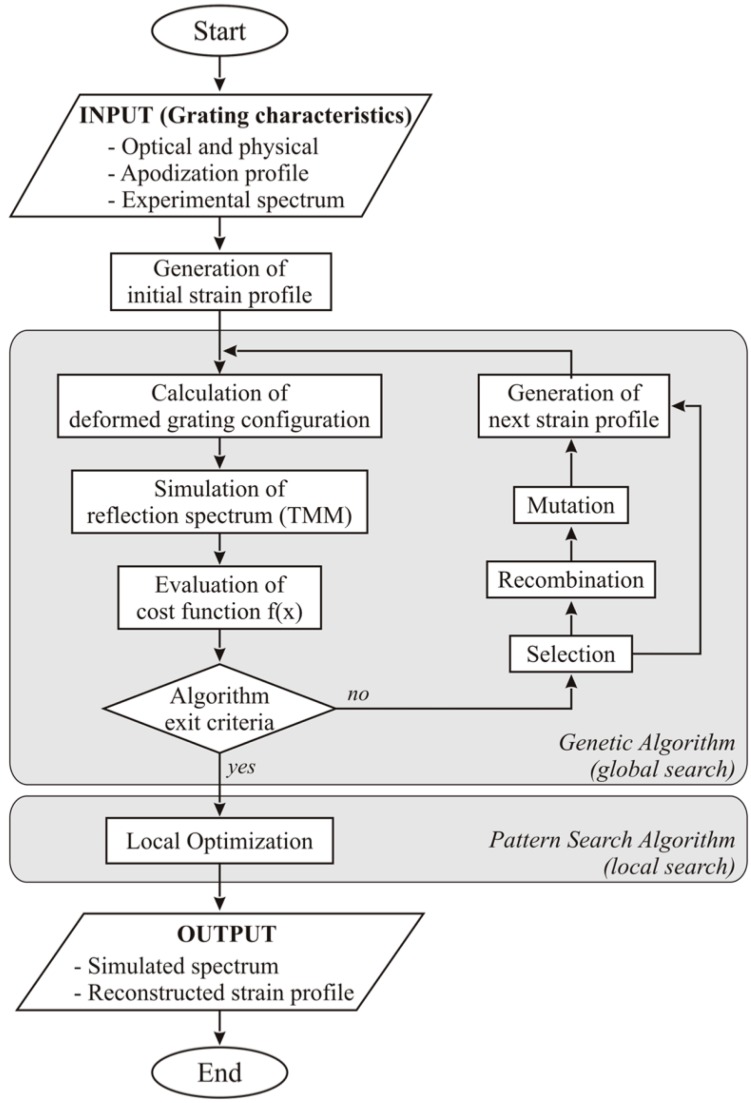
Method for spectral reconstruction proposed by Bettini et al. based on CFBG spectral analysis. Image from [57].

Bettini et al. [57] in 2015 proposed a method for spectral reconstruction that is illustrated in Figure 8, which is based on two rounds of optimization; the method is applied for strain sensing. The algorithm starts by inputting the optical parameter of the grating (start/end wavelength, chirp rate, strain sensitivity) and the physical dimensions of the grating. These parameters are set in reference condition (with the initial strain value reported as a reference). Based on these parameters, by means of the CFBG model (in [57] the model is accounted based on the transmission matrix model, TMM), it is possible to analytically derive the optical spectrum of the CFBG.

At each measurement time, the algorithm generates a new strain profile, that is then implemented in the TMM extracting a new simulated CFBG spectrum. A cost function is then evaluated, comparing the TMM-simulated spectrum with the measured spectrum. The format of the cost function (CF) elaborated in [57] is the following:

(14) $$CF=W_{sp}E_{sp}+W_{\lambda C}E_{\lambda C}+W_{FWHM}E_{FWHM}$$

and is composed of three terms (*E_sp_*, *E_λC_*, and *E_FWHM_*), each one multiplied by its own weight coefficient (*W_sp_*, *W_λC_*, and *W_FWHM_* respectively). The three terms are explained as follows: the term *E_sp_* contains the sum of the difference between the simulated and measured spectrum calculated at each wavelength; the term *E_λC_* refers to the difference in central wavelength, measured as in Equation (10) between the measured and simulated spectrum; the final term *E_FWHM_* refers to the difference in FWHM bandwidth between measured and simulated spectrum, evaluating the FWHM as in Equations (11)–(13). At this step, two optimizations are used. The first one is a genetic optimization algorithm that aims at a first minimization of the CF, while the second algorithm implements a local optimization to further refine the strain profile. For each measurement, the strain profile that minimizes the CF is the optimum strain profile, and the best estimation of the strain profile that the spectral reconstruction returns.

The work from Bettini et al. studies three typologies of strain: spatially uniform strain, linear gradient as in Equation (9), and linear gradient with a gradient change, that can be evaluated as:

(15) $$\Delta \varepsilon \left(z\right)=\{\begin{array}{cc} \varepsilon_{01}+\varepsilon_{11}z & 0\leq z\leq L_{1}\\ \varepsilon_{02}+\varepsilon_{12}z & L_{1}<z\leq L \end{array}$$

constrained to the continuity of strain in the point *L*_1_. This method allows reducing the complexity of estimating the strain on all *M* grating slices, to 4 parameters in Equation (15). A similar method has been applied by Palumbo et al. [36] using a TMM model, detecting a temperature variation.

An alternative method with a simpler and faster implementation, potentially suitable for real time computation as it is not based on a genetic algorithm, has been proposed by Korganbayev et al. in 2018 [35]. This technique is based on the following algorithm, similar to Figure 8 in flow-chart but with some differences in the initialization and in the optimization, applied to a Gaussian temperature pattern estimation as in Equation (8):

1. Initialize a CFBG model based on CMT [7]. The parameters input to the model are: *L*, *L_g_*, *δn_eff_*, *n_eff_*, *kL_g_*, *λ_B_*(0). The parameters can be obtained from the CFBG manufacturer, or estimated as in [72]. The simulated spectrum of the CFBG is labeled as *R_SIM_*(*λ_i_*), *i* = 1, 2, …, *N*. The wavelength grid *λ_i_* is defined by the detector.
2. Calibrate the CFBG using a thermal bath, obtaining the temperature sensitivity *s_T_*.
3. Measure the CFBG in reference condition, obtaining the spectrum *R_MEAS_*(*λ_i_*).
4. Obtain a digital equalization filter *H*(*λ_i_*) such that *R_SIM_*(*λ_i_*) × *H*(*λ_i_*) = *R_MEAS_*(*λ_i_*) in reference condition.
5. For each measured spectrum:
  - Generate a temperature pattern using Equation (8).
  - Apply the temperature pattern to the CMT model, obtaining a new *R_SIM_*(*λ_i_*).
  - Equalize the simulated spectrum using the previous function *H*.
  - Calculate the cost function CF, as the root mean square error (RMSE) between the equalized simulated spectrum and the measured spectrum.
  - Repeat this cycle, varying the parameters in Equation (8), until the CF is minimized.
6. The set of parameters that minimizes the CF is considered to be the best estimation of the temperature profile.

The CF can be expressed, in formula, as the RMSE:

(16) $$CF=\sqrt{\frac{1}{N}\sum_{i=1}^{N}{\left[R_{MEAS}\left(\lambda_{i}\right)H\left(\lambda_{i}\right)-R_{SIM}\left(\lambda_{i}\right)\right]}^{2}}$$

This system allows three simplification with respect to [57]. In first place, the cost function can be calculated directly from the spectra, without the need to perform additional calculations. In second place, by implementing an equalizer it is possible to skip the apodization pattern estimation that is part of the initialization of Figure 8. Since the grating maintains its apodization pattern during the thermal or strain shifts, the equalizer can compensate for the ripples or the change of reflectivity. Lastly, in [35] the optimization is based on two fast steps: an iterative algorithm provides an initial estimate of the three parameter set (*T*_0_, *z*_0_, *v*); then, a Monte Carlo algorithm [73] refines the estimate.

The spectral reconstruction methods are overall tied to the assumption that the temperature or strain pattern has a shape known a priori over the active grating length. On this side, they require a harder implementation than distributed sensors [42,43]. However, they have a faster, smaller, and less bulky optical hardware and they allow a precise control of the sensing region at the sub-millimeter scale [45].

## 5. Applications of CFBG Sensors

In this section, the main applications of CFBG sensors are reviewed. In Table 2, a list of applications is reported, outlining the case scenario, the main CFBG parameters, and the detection system. It is possible to classify the case scenarios of CFBG sensors in the following classes of applications, whereas the inherent spectral characteristic of the CFBG and the capability of detecting profiles is a key asset:

- Sensors for temperature measurement in thermal ablation [35,36,38,74];
- Positioning detection of localized heat source [40];
- Strain measurement in structural health monitoring [57];
- Detection of damages on CFRP (carbon-fiber reinforced polymer) [41,75,76];
- Measurement of velocity of shock waves [34,77];
- Monitoring of transmission lines [78];
- Detection of liquid containers inclination and level [53,79,80,81];
- Localization and estimation of high-pressure events [82];
- Distributed strain and temperature measurement [83,84,85];
- Hybrid sensors [86,87].

### 5.1. Monitoring of Minimally Invasive Thermo-Therapies

The most consolidated and mature application of CFBG sensors in the current scenario is in the real-time temperature sensing for medical thermal ablation in interventional cancer care [88,89]. Thermal ablation has a consolidated usage in correction of cardiac arrhythmia, pain management, and electrical resections, but a trend of the latest two decades is the application of thermal ablation for the minimally invasive treatment of tumors [90,91,92], after being diagnosed and localized. The physical driver for thermal ablation is identified in high temperature recorded in the tumor tissue [90], namely the thermal dose. According to Sapareto and Dewey [92], temperatures higher than 42–44 °C are toxic for cells, and a reference condition for clinical use is the exposure to 52 °C for >60 s. For fast ablation phenomena [88,89,90], the threshold for operation is 60 °C: at this temperature, the rapid coagulation of proteins guarantees a nearly instantaneous mortality of tumor cells in the tissue. The goal of thermal ablation is to deliver in the tissue, in a mini-invasive procedure, a highly selective thermal field, that exposes the whole tumor to ≥60 °C while avoiding damages to the healthy tissue.

In thermal ablation, electromagnetics principles are use to deliver the energy to the tissue through a percutaneous applicator with minimal invasiveness and compact form factor [93,94,95], or a non-contact device [91]. Radiofrequency ablation (RFA) makes use of a RF generator (450 kHz) to deliver a confined heat field in tissues having impedance of 60–150 Ω [74,89]. Microwave ablation (MWA) uses a 2.4 GHz source coupled to a miniaturized antenna [90]. Laser ablation (LA) uses a solid-state or fiber laser coupled into a large-core fiber inserted in the tissue [35,38]. HIFU (High-intensity focused ultrasound) is the only type of non-contact ablation, and makes use of an array that focused ultrasound delivery to a spot-size tissue [91]. In percutaneous ablation [88], the miniaturized applicator is inserted by the clinician to the center of tumor; after turning on the RF, MW, or laser source, the tissue starts heating in proximity of the applicator, and heat progressively transfers to the peripheral sides of the tumor ablating a volume of tissue that corresponds to the size of the tumor and a safety margin.

For an efficient thermo-therapy, it is necessary to monitor the temperature in situ [88] in order to have a correct detection of the amount of tissue ablated in each moment of the ablation. In [88,89], it is observed that this measurement is complicated by the harsh environment and by the specific technological features of the sensor requirement. The sensor must operate in contact with the tissue, thus a good tensile strength, mechanical resistance, and biocompatibility (ISO 10993 [96]) are required. The sensor also operates in proximity of a high electromagnetic field, thus it is necessary that is immune to electromagnetic radiation. Sensors with metallic packages are not desirable, as they can direct a part of the high-intensity radiation towards the sensor rather than towards the tissue, reducing the efficiency of ablation (particularly RFA). Most importantly, the sensor(s) must render a thermal map (temperature as a function of space and time) that is compatible with gradients as high as 5 °C/mm in space and 0.5 °C/s in time [88,89]. The first generation of fiber optic sensors applied to medical thermo-treatments was based on fluoroptic sensors [97,98]; however, these probes are able to measure only a single-point temperature. The first application of multi-point sensors in RFA has been reported in [10], using an array of five standard FBGs having 5 mm length and 10 mm sensor spacing. The work by Macchi et al. [99] improved the spatial resolution by using an optical backscatter reflectometer [43], reducing the spatial resolution below the millimeter. A chirped FBG, for this application, appears as a better practical solution than both FBG and OBR: while maintaining the capacity to resolve the temperature at the millimeter scale or below, it is based on an interrogator significantly faster and more inexpensive than an OBR, and the data readout is much simpler.

**Figure 9:**
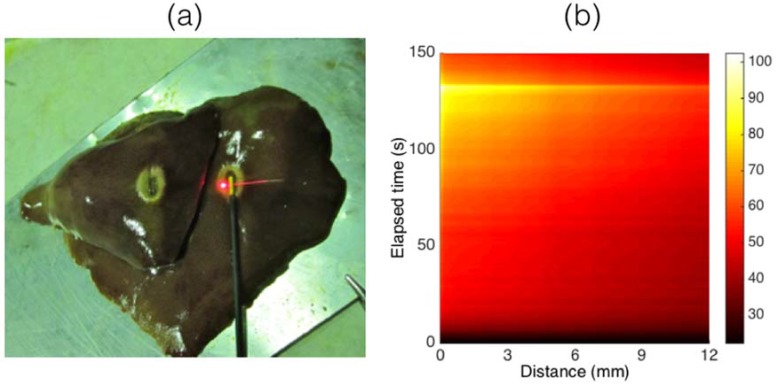
CFBG for in situ temperature detection in RFA; image adapted from [89]. (**a**) Positioning of the CFBG in the ablated tissue; (**b**) example of a measured thermal map (the colorbar reports temperature in °C).

The first work that demonstrated the use of a CFBG in RFA has been reported by Tosi et al. [74] in 2014, and main results have also been reviewed in [88,89]. In this work, a single CFBG with a linear chirp, having 15 mm length and 33.4 nm FWHM (Technica S.A., Atlanta, GA, US) inscribed on a SMF fiber, has been used as a temperature sensor. The experiments in [74] are based on a RFA single-tip applicator powered by a 450 kHz medical grade generator. Ablations have been performed on ex vivo porcine tissue. In Figure 9, adapted from [89], the main experiment outline is shown. Figure 9a shows the insertion of the CFBG in the tissue; the CFBG is coated with an acrylate jacket, which is illuminated in the Figure. The length of the sensing region is 15 mm, and is transverse to the applicator; as suggested by the picture, the ablated tissue is small in size, with a longitudinal extension slightly over 1 cm. In Figure 9b an example of thermal map of [74] is reported. The temperature rises in proximity of the tip with a quadratic trend, slowing the increase rate after 50 s, until the peak temperature is reached at 132 s. At this moment, when the tissue temperature reaches and slightly exceeds 100 °C, the impedance quickly rises and the generator discontinues the power, causing the tissue to rapidly cool. The analysis of CFBG spectra in [74] is based on a simple algorithm, that compares the left and right spectral edges and the center of the spectrum, from the initial reference measurement. This approach is effective for a spatially monotonic temperature, as for the experiments in [74].

A subsequent study by Saccomandi et al. [38] in 2017 reported the application of linearly chirped FBGs in laser ablation. This study aims at the analysis of the thermal gradients observed during experiments of laser ablation in cancer care [88]. The analysis carried out on CFBG grating spectra aims at the evaluation of the central wavelength and the FWHM of the reflection spectrum, measured with a scanning-laser interrogator [54]. A CFBG having 10 nm FWHM and 15 mm length has been used in the experiments to detect the in situ thermal pattern during a laser ablation procedure, performed with a Nd:YAG 1064 nm solid-state laser coupled to a large-core fiber. The authors presented the results in three case scenarios: (1) in presence of a linear gradient along the grating, showing that both the central wavelength and the FWHM increase linearly as the gradient increases; (2) performing LA on ex vivo animal model, using as a reference an array of FBGs; (3) performing in vivo tests on an animal (anesthetized male pig) in order to prove the effectiveness of CFBG sensing.

A further step towards the use of CFBGs in thermal ablation has been performed by Korganbayev et al. [35] and Palumbo et al. [36]. In [35] the method of spectral reconstruction is introduced to a laser ablation pattern, using a CMT model for the grating based on discrete Erdogan’s theory [7]. This method has also been validated using a linear gradient, and a solid-state laser ablation system. The work of Palumbo et al. [36] makes use of a transmission matrix model of the CFBG [6] and a multi-tip laparoscopic device for RF ablation [100] in order to detect the thermal pattern in proximity of the laparoscopic device (Habib 4× Laparoscopic Bipolar Resection Device [101]). The main improvement of [35,36] over [38,74] is the application of spectral reconstruction method, that allows detecting thermal pattern having a Gaussian shape typical of RFA and LA [88,89,90,93].

To date, the implementation of spectral reconstruction in thermal ablation is one of the main milestones for the application of CFBGs in thermal pattern measurement in real time in thermal ablation, with possibility of outperforming FBG sensors in terms of spatial resolution [45,87].

### 5.2. Localization of Spot-Size Heat Source

The work of Nand et al. [40,102] can be considered a predecessor of the application of CFBG in thermal ablation, as it introduces the detection and localization of spot-size heat sources on the grating length. In this study, a 15-mm CFBG inscribed on a H_2_-loaded SMF and having a relevant chirp rate (1.89 nm/mm) has been used as a sensor, detecting the temperature on a white light setup. The experiments have been carried out using a hot-wire, placed along the grating and having a hot-spot thermal shape with 50 °C amplitude and approximately 1 mm of standard deviation.

In case of a hot-spot temperature change, similar to a Gaussian function as in [35] but having a fixed amplitude and standard deviation, Nand et al. [40] developed a method of detection based on the Fast Fourier Transform (FFT) of the normalized spectrum of the CFBG. The main task of this work is the estimation of the hot-wire position, which is carried out with root-mean square (RMS) accuracy of 0.03 mm over the 15-mm grating length.

### 5.3. Structural Health Monitoring

The work of Bettini et al. [57] proposes the use of CFBG sensors in the determination of multi-point strain profiles in structural health monitoring by means of a spectral reconstruction technique, providing a valuable alternative to the distributed sensing methods [103] or transverse load estimation by polarization analysis [104].

**Figure 10:**
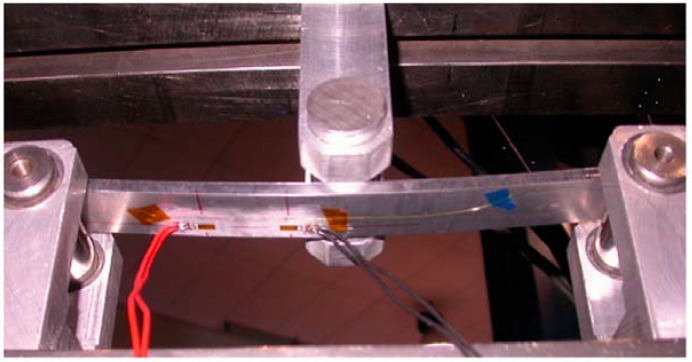
Photograph of the CFBG embedded in a load system for 3-point strain detection proposed by Bettini et al. Image from [57].

In [57] a spectral reconstruction technique has been used to detect a strain having expression as in Equation (15), based on a two-step initialization (determination of grating parameter, and estimation of apodization profile), and a two-step optimization (genetic algorithm followed by an iterative optimization step to further refine the coefficients estimate). The reported system has been tested using a 3-point strain system shown in Figure 10 (image from [57]) that applies a load in a calibrated location within the central region of the CFBG while maintaining fixed the edges.

The work from Bettini et al. [57] shows an important path for the use of CFBG sensors in structural health monitoring (SHM), whereas the grating is embedded in structures or smart structures [5,105] for real-time strain distributed sensing.

### 5.4. Monitoring of CFRP Damages

Carbon fiber reinforced polymer (CFRP) materials are used in aerospace and automotive, as they combine resistance, tensile strength, and lightweight [76,106,107]. As CFRP elements are often used as load-bearing elements, particularly in aerospace [41], it is important to introduce a sensing system that is able to provide an early-detection of CFRP damages. Fiber optic sensors based on FBGs have been introduced for the detection of damages of CFRP, by embedding the sensors on a CFRP laminate; notable works of Zhou et al. [106] and Yashiro et al. [107] show the possibility of using an FBG or FBG array for the detection of strain in laminated CFRP.

The possibility of detecting strain events along the spectrum of the CFBG [108], exploiting the inherent capability of the chirped grating to spatially resolve a strain pattern has been explored, most notably, by Takeda et al. [76] for monitoring CFRP delamination, by Yashiro et al. [41] for monitoring of online damages of CFRP, and by Yandy et al. [75] for detecting the position of damages on CFRP.

The work of Yashiro et al. [41] focuses on the detection of the formation of damages of CFRP holed laminates using an embedded CFBG, having a long length (30 mm) and a FWHM of 8 nm. The simulations reported in [76,107] show that in the event of a delamination, result in a strain applied through the CFBG length, having a peak around the delaminated region. The damage analysis show that the grating changes spectrum in the correspondence of the region exposed to the damage, observing a spectral discontinuity that depends on the location of the damage.

The investigation by Takeda et al. [78] makes use of a CFBG having length of 50 mm and low chirp rate (0.1 nm/mm, corresponding to 5 nm FWHM), embedded in a CFRP laminate. The scope of this work is to provide a model and an experimental methodology to detect the formation of delamination of the CFRP composite. In this study, a CFBG was embedded in a CFRP laminate of 70 mm length, that is progressively delaminated on its edge. The result is a strain linear gradient along the CFBG direction, which is detected in a strain event that compresses the spectral width of the CFBG.

A similar work has been reported by Yandy et al. [75], measuring and localizing the position of defects on a CFBG laminate with a weakly chirped FBG (1 nm FWHM). In this work, the analysis of CFBG spectra is paired with the group delay analysis.

Overall, the CFBG technology is effective on the detection of CFRP composites of short length, by embedding the grating into the material with a suitable connector [5]. CFBGs compete with distributed sensors [42,43,103] that enable detection of strain on a longer region. In multiple applications, the availability and multiplexing capability offered by standard FBG arrays [5,104,105,106] provides a more straightforward implementation in aerospace, whereas the possibility to have a larger sensing network using time- and wavelength-division multiplexing, and increasing the distance between each sensing point, is a key factor [109].

### 5.5. Measurement of Shock Wave and Detonation

Whereas the previous works perform a detection of the grating spectra and a subsequent analysis at a relatively slow speed, it is possible to use CFBG spectral variations in order to detect the shock wave propagation related to detonation. The studies presented by Rodriguez and Gilbertson [77] and Wei et al. [34] in 2017 implement a fast-detection system for CFBG sensor during shock waves.

The system reported in [77] is based on the measurement of the central wavelength and FWHM of the CFBG, in a similar fashion to [38], using a high speed photodetector. This work follows a set of preliminary studies within 2013–2015 [110,111,112,113], consolidating the approach based on a high-speed white-light system [108] as well as a time-streaked spectral detection system [77,111]. The experiments reported in [77] are reported for the tracking of a plastic bonded explosive, for radially decaying shock, and for a shock wave tracking of an aluminum cylinder, reporting case scenarios typical of detonation science [110,111] as well as high-energy physics [112].

**Figure 11:**
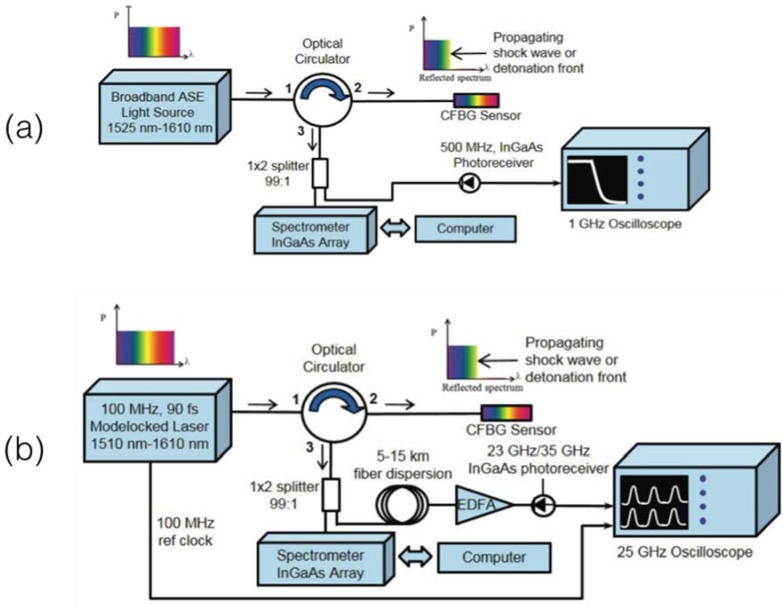
Schematic of high-speed CFBG interrogation systems proposed by Rodriguez and Gilbertson: (**a**) InGaAs photodetector-based setup; (**b**) fs laser-based scanning setup. Image adapted from [77].

The principle of operation of both systems, shown in Figure 11 (image adapted from [77]) is that when the CFBG is exposed to a detonation or shockwave event, the FBG length progressively reduces due to the intense shock. The velocity at which the CFBG gets shortened is corresponds to the speed of detonation, and is proportional to the speed at which the FWHM of the grating gets reduced. The setups assembled by Rodriguez and Gilbertson both aim at the real-time analysis (with tens of nanoseconds time resolution) of the FWHM reduction of the bandwidth. At the start of the experiment, the grating has the highest FWHM; through the fast detonation event, the grating gets progressively damaged, at a linear rate, and its bandwidth reduces until the whole CFBG spectrum is depleted. By measuring the spectrum of the CFBG at high speed, it is possible to resolve the detonation speed by dividing the CFBG length by the time recorded for the whole CFBG spectral depletion.

Two setups illustrated in Figure 11 have been used in [77,112,113]. The first setup, in Figure 11a is based on a broadband ASE source connected to the CFBG through a circulator. The output power is collected by a high-speed InGaAs photodetector, and recorded by an oscilloscope and acquired through a data acquisition (DAQ) system. Assuming the CFBG spectrum to be flat, and the source to have a flat spectrum in the region corresponding to the grating, the output power collected at the photodetector is proportional to the FWHM of the grating: this approach is sensitive to power drifts, that can occur during the detonation event, but provides a high speed, limited only by the photodetector.

The second setup, in Figure 11b, namely time-streaked spectrally resolved approach, is based on a fs laser (90 s) pulse duration with 10 ns repetition time (100 MHz repetition rate). The scanning laser is connected to the CFBG through a circulator, and the output is amplified by an erbium-doped fiber amplifier (EDFA) after a ~10 km dispersive fiber expands the pulse width. The output power is collected by an InGaAs photodetector. The scanning laser system allows recording, at a sufficient speed (limited to 10 ns by the repetition rate of the laser) the whole CFBG spectrum allowing a more accurate detection of the CFBG bandwidth, that does not depend on spectral ripples of the ASE source and CFBG.

The results in [77] show the measurement of the detonation velocity implemented with several CFBGs having length that ranges from 10 mm to 200 mm, and chirp rate from 0.35 nm/mm to 3.45 nm/mm, with acrylate and polyimide coating. The velocity of plastic explosive detonation has been measured as 8840 m/s.

The work by Wei et al. [34] follows the same working principle, consisting in the measurement of destructive events occurring to the CFBG by measuring the change of FWHM in the time domain. The authors implemented a system based on a high-speed photodetector that returns an output power proportional to the CFBG bandwidth. The main difference with respect to [77] is that the probe is designed with 2 channels, each consisting by one CFBG each having length of 40 mm and FWHM 30 mm, approximately.

**Figure 12:**
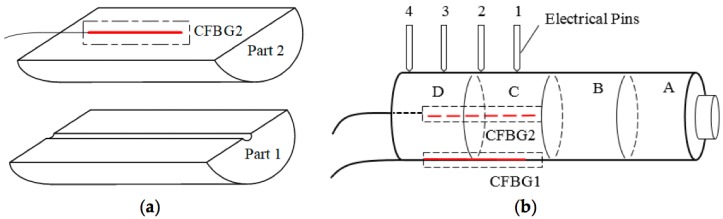
Schematic of the CFBG assembly proposed by Wei et al. (**a**) Assembly of top and bottom parts of the probe; (**b**) structure of the whole sensing probe, inclusive of reference pins. Image from [34].

The two-sensor system assembly is shown in Figure 12 (image from [34]). The experiments in [34] show a detonation speed of 6011 m/s and 6147 m/s, with a difference up to 1.8% with respect to the electric pins used as a reference in the measurement.

With respect to the previously reported applications, whereas the CFBG spectrum is subjected to a relative small deformation due to a spot-event of a linear- or Gaussian-shaped pattern, in shock wave and detonation speed detection the CFBG has an abrupt variation of the bandwidth, that progresses from the full spectrum to a completely depleted reflection spectrum in few microseconds. The use of a CFBG, in lieu of a uniform FBG, allows the detection of the FWHM which can be implemented using the setups in Figure 11.

### 5.6. Monitoring of Power Transmission Lines

The work of Wydra et al. [78] proposes the use of CFBG sensors with a linear chirp in the context of real-time estimation of parameters of overhead transmission lines (OTL). The problem of the determination of the damages and failures to transmission lines carrying high power rates has been addressed using imaging-based and electromechanical technologies [114,115]. Fiber optic sensors however have significant advantages with respect to other inspection methods based on imaging or mechanical sensors [116,117]. Most importantly, the low loss of optical fibers (about 0.2 dB/km for single-mode fibers) allows positioning the sensing point at a long distance from the interrogator, giving the opportunity to extend the coverage of the detection area, while at the same time the electromagnetic immunity of glass fibers allows positioning the sensor in close contact with the transmission line, having no electromagnetic compatibility problems. In addition, the dual sensitivity of FBGs to strain and temperature allows creating sensing networks for outdoor operation, whereas two sensors can be used: as in [117], it is possible to have an FBG-based strain sensor to perform an early diagnostic of the transmission line, while a second FBG is mounted loose and compensates the temperature effects.

**Figure 13:**
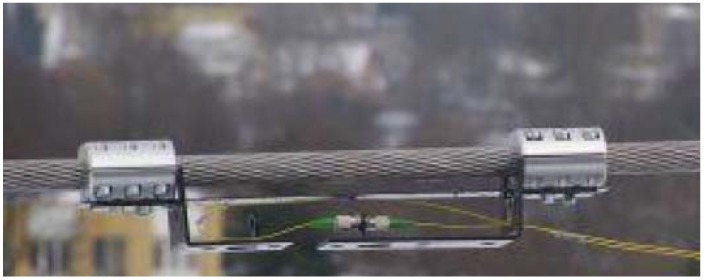
CFBG for overhead transmission line sag detection reported by Wydra et al. Image from [78].

In [78], the OTL diagnostic is proposed using one CFBG, rather than uniform gratings. The system by Wydra et al. is shown in Figure 13 (image from [78]): the sensor is a linearly chirped FBG with 1.7 nm FWHM and a 0.1 nm/mm low chirp rate, clamped on a 110 kV OTL. The system makes use of a plate to store the fiber connectors in proximity of the grating.

Due to the outdoor measurement, the CFBG is subjected to both strain (due to power line sag) and temperature variations. The results in [78] show the possibility to discriminate the two effects. Temperature changes result in a variation of the central wavelength of the CFBG, but do not affect the FWHM of the grating. Instead, the power line sag results in an elongation of the CFBG that results in an expansion of the FWHM: the authors report a linear increase of the FWHM of 6.03 nm/mm reported as increase of FWHM per each mm of elongation of the power cable, while the residual cross-sensitivity to temperature is 0.1 pm/°C, that can be further compensated by measuring the shift of the central wavelength.

Overall, the method reported by Wydra et al. is effective in the diagnostic of power cables potential failure, as it is able to fully discriminate the strain effects (encoded in the FWHM) from the temperature effects (encoded in the Bragg wavelength).

### 5.7. Precision Detection of Liquid Containers Level and Inclination

By shrinking the diameter of the cladding of an FBG, it is possible to increase the sensitivity of the grating to the external refractive index [25]. This type of grating, labeled etched FBG (EFBG) or exposed FBG or thinned FBG, is an interesting device for biosensing [118]. The principle of operation is that, by wet- or dry-etching a significant portion of the fiber cladding, the confinement factor of the optical fiber changes, introducing a change of the effective refractive index, resulting in a change of Bragg wavelength of the grating [24]. If the etching is significant, and the cladding diameter is etched from the initial ~125 μm to few tens of μm [25], the effective refractive index has a strong dependence on the external refractive index. As a consequence, the EFBG Bragg wavelength depends on the outer refractive index, with a sensitivity ranging from few pm/RIU (refractive index units) to several nm/RIU as a function of the cladding thickness [24]. In biosensors [25,118], it is a common practice to etch uniform gratings and measure the change of Bragg wavelength [24], functionalizing the fiber tip by means of bioreceptors [23,118].

Since an etched grating introduces a dependence of its properties on refractive index, an etched CFBG, following Equations (1)–(3) introduces a dependence on the refractive index of each grating slice, similarly to a temperature pattern estimation. Whereas in the development of a biosensor this can act as a complication, as the refractive index events can be encoded in any part of the grating (despite the recent work of Du et al. [119] opens interesting avenues for distributed refractive index sensing), it is possible to use an etched CFBG for the detection of step-changes of refractive index along the grating, namely for the detection of liquid level.

This principle has been exploited by Chang et al., which made use of an etched CFBG for the precision detection of liquid level in a vertical configuration [80], and subsequently for a bi-dimensional system for inclination detection [53,79]. In these works, the main principle of operation is the localized variation of the spectrum observed in the CFBG in correspondence of the transition between air and liquid (e.g., water) as the CFBG is partially immersed in liquid.

The liquid-level indicator reported in [80] is based on a single CFBG (9.3 nm bandwidth, 7 mm length) etched in a 20% solution of hydrofluoric acid (HF) until the cladding thickness is reduced to 12 μm. The detection system based on an ASE source and an optical spectrum analyzer (OSA) detects the spectrum of the CFBG. When the grating is partially immersed in water, the spectrum of the grating changes as the longest wavelengths are in air while the shortest wavelengths are held in water, creating an overlapping portion of the spectrum. By detecting the center of the overlapping part of the spectrum, and measuring its wavelength shift, it is possible to estimate the width of the grating portion immersed in the liquid. The results by Chang et al. in [80] show a sensitivity of 1.21 nm/mm, that corresponds in a good degree of approximation to the chirp rate of the grating (1.32 nm/mm).

**Figure 14:**
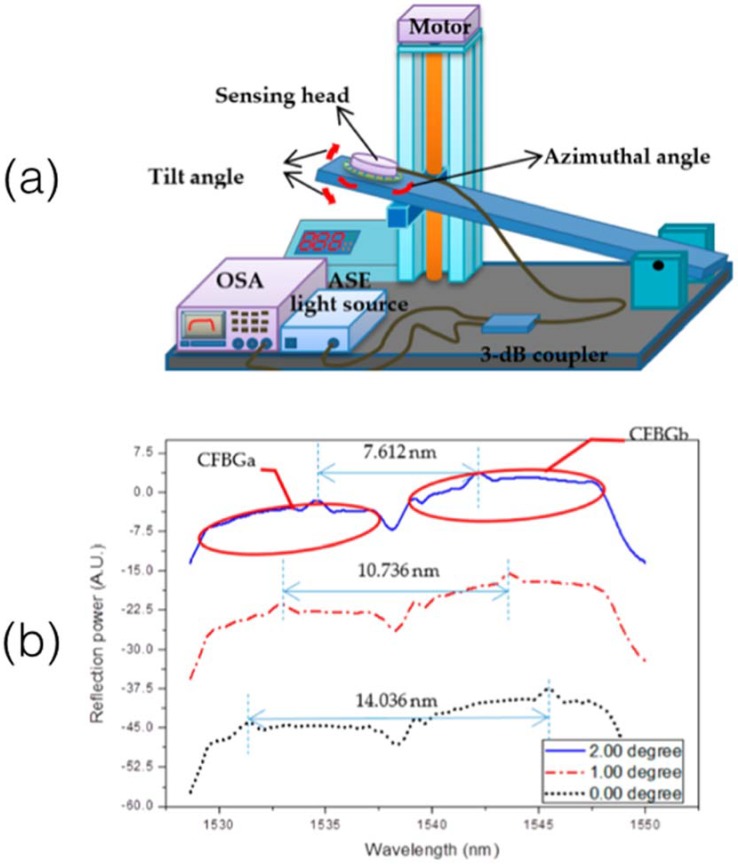
2D inclinometer reported by Chang et al. based on a pair of CFBGs. (**a**) Schematic of the setup; (**b**) working principle. Image adapted from [53].

In two subsequent works [53,79] Chang et al. extended these results to a bi-dimensional (2D) inclination system, capable of measuring the tilt in xy directions for a grating along the axis *z*. The principle of operation is shown in Figure 14 (image adapted from [53]). Two CFBGs compose the sensing head, connected in cascade and mounted perpendicularly from each other in the probe. The pair of CFBGs have similar parameters (FWHM 8.4 nm, length 7 mm, diameter of cladding 12 μm), and are connected in cascade as shown in Figure 14b. Similarly to [80], the spectrum of each CFBG exhibits an overlap that is directly related to the portion of the CFBG immersed in liquid. By measuring the offset between the spectral overlap of the first and the second CFBG, it is possible to detect the tilt angle, obtaining a 2D inclination system that works similarly to a bubble.

A method for inclination and tilt angle detection, alternative to a fully etched CFBG, has been proposed by Osuch et al. [81]. This architecture is based on a two-sided tapered CFBG, in which the two sides of a 20-mm CFBG have the larger cladding thickness, while the central part is fully tapered. At different inclination angles, the spectrum of the two sides of the CFBG changes, leaving a broad-band region in which the average reflectivity is strongly dependent on the inclination angle, while temperature variations result in a shift of the overall spectral envelope.

The methods reported by Osuch [81,84] are based on the inscription of a dual-tapered CFBG, which appears as a two-sided grating on a counter-tapered fiber [120,121,122]. This configuration allows changing the spectrum of the CFBG(s), as a function of the tapering pattern.

### 5.8. Localization and Estimation of High-Pressure events

Swart et al. [81] proposed in 2005 a configuration based on CFBG for the detection of the positioning of high-pressure events along the grating length. The system of Swart et al. is based on a polymer-coated CFBG having a long length (100 mm) and a short chirp rate (0.011 nm/mm, corresponding to a FWHM of 1.1 nm).

The interrogation system is based on a tunable laser modulated by a Mach-Zehnder Modulator by a RF tone (500 MHz). A circulator routes the light source to the sensing CFBG, and the reflected component is detected with a phase photodetector amplified by an EDFA. This configuration allows detecting the phase component of the CFBG spectrum.

When a force is exerted on a specific location of the CFBG, in a single stress point, a change of phase is observed; the peak of the phase signal has an amplitude proportional to the force exerted on the grating, while its wavelength shifts according to the location of the force. The response in phase to the location of the pressure event is linear, with coefficient 0.0116 nm/mm that corresponds almost exactly to the CFBG chirp rate; the amount of phase change has a non-linear dependence on the force exerted on the grating, and varies from 0 rad to 0.3 rad when the mass applied to the grating changes from 0 g to 55 g. This methodology has also been conceptualized in [123] for the esophageal pressure detection.

### 5.9. Distributed Measurements

The works reported by Liu et al. [83] in 2011 and Wang et al. [82] in 2015 implement distributed sensing [42,43] using a CFBG as a distributed weak reflector, in a configuration similar to optical frequency-domain reflectometry. The system reported in [83] focuses on the detection of strain, and is based on a distributed feedback laser modulated with a chirped waveform. The sensor is based on two identical CFBGs having the same linear chirp, one acting as a reference and one as a strain sensor. The local strain values are detected by analyzing the beat frequency at the photodetector. The system reported in [82] makes use of a similar working principle, but the CFBG is inscribed on a highly birefringent fiber in order to discriminate strain and temperature [124]. Overall, the results investigated in [82,83] show the capability of the CFBG to act as a distributed reflector on a length of 17 mm [82] to 115 mm [83]. A similar work was proposed by Osuch et al. [84] for strain measurement, using a dually tapered chirped FBG.

### 5.10. Hybrid Sensors

CFBGs can be used in conjunction with other fiber optic sensors operating on the same bandwidth, and interrogated by the same setup, in order to enable multi-parameter detection or thermal compensation [84,85,125]. Sun et al. [84] reported a refractive index sensor that combines a long-period grating (LPG) [7] with a CFBG having 20 mm length and 16 nm bandwidth, in order to introduce a highly sensitive refractive index detector. The LPG and the CFBG devices couple cladding modes on a wide bandwidth, resulting in a CFBG spectrum that is perturbed by the cladding modes introduced by the LPG, that appear as a spectral hole. When the refractive index is changed, the relative position of the spectral hole introduced by the LPG in the spectrum shifts, according to the amount of RIU changes.

Duraibabu et al. [85] demonstrated a dual pressure-temperature sensor with spatial resolution capability by merging a pressure-sensing extrinsic Fabry-Perot interferometer (EFPI) with a CFBG having 3 nm FWHM. The device is fabricated by fiber splicing on the tip of a CFBG. The EFPI appears as a broadband low-finesse interferometer, while the CFBG appears as a high-reflection component in the spectrum.

## 6. Conclusions

In conclusion, chirped fiber Bragg grating sensors and their applications have been reviewed in this work, highlighting their main features, emerging trends, and the case scenarios in which the characteristics of CFBGs are a key performance factor. This review focuses on the specific use and features of chirped FBG, in particular with a linear chirp, as this type of sensor differentiates from standard uniform FBGs.

CFBGs can be considered semi-distributed sensors: like standard FBG they have an active length in which the sensing phenomena can be accounted, but like distributed sensors their spectrum depends on the strain or temperature pattern across the whole grating length. The spectral reconstruction method is becoming a consolidated routine to evaluate strain and temperature patterns at the millimeter scale.

The main applications of CFBGs exploit the distributed sensing features on a small scale. The main applications are in real-time temperature profiling in thermo-therapies, structural health monitoring and composites monitoring, and measurement of detonation speed. However, the possibility of combining the CFBG with other fiber optic sensors, or to modify the fiber profile (changing the fiber compound or exposing the fiber by means of chemical etching) can introduce advanced sensing features.

Future directions will be addressed to improve and standardize the spectral reconstruction method, in order to simplify the detection using commercial off-the-shelf interrogators, affordable for most applications.

## Figures and Tables

**Table 1:** Reports of inscription of CFBG in conventional and specialty fibers and formats.

First Author	Ref.	Fiber Type	Inscription Method	CFBG Length	FWHM	Chirp Rate	Sensitivity
Standard CFBGs							
Korganbayev	[35]	SMF	Phase mask	50 mm	40 nm	0.8 nm/mm	10.2 pm/°C
Korganbayev	[35]	SMF	Phase mask	15 mm	20 nm	1.33 nm/mm	10.2 pm/°C
Saccomandi	[38]	SMF	Phase mask	15 mm	10 nm	0.67 nm/mm	10.4 pm/°C
Palumbo	[36]	SMF	Phase mask	45 mm	56 nm	1.24 nm/mm	~10 pm/°C
Nand	[40]	SMF, H_2_-loaded	Argon-ion laser	15 mm	28.4 nm	1.89 nm/mm	~10 pm/°C
Bettini	[57]	SMF	Phase mask	30 mm	45 nm	1.5 nm/mm	
Specialty CFBGs							
Marques	[58]	PMMA step-index	KrF laser, phase mask	25 mm	3.9 nm	0.16 nm/mm	−131 pm/°C 1.77 pm/με
Min	[37]	BDK-doped POF	KrF laser, phase mask	10 mm	0.2–1.2 nm		−56.7 pm/°C 0.71 pm/με
Idrisov	[59]	Birefring. SMF	Excimer laser, draw tower	5 mm	0.5 nm	0.1 nm/mm	12.3 pm/°C
Voigtlander	[62]	SMF	Ti: sapphire, tunable mask	20 mm	Up to 2 nm		
Xiao	[60]	Tapered MMF	Excimer laser, phase mask	3.5 mm	5.5 nm	1.57 nm/mm	−5.2 nm/RIU
Qiao	[63]	SMF regener	Excimer laser, phase mask	20 mm	26.3 nm	1.31 nm/mm	15.1 pm/°C
Bernier	[61]	SMF	Ti:sapphire, phase mask	25 mm	85 nm	3.4 nm/mm	
Bernier	[61]	SMF, H_2_-loaded	Ti:sapphire, phase mask	25 mm	206 nm	8.24 nm/mm	
Bernier	[61]	SMF, H_2_-loaded	Ti:sapphire, phase mask	35 mm	310 nm	8.85 nm/mm	

**Table 2:** Review of applications of CFBG sensors.

First Author	Ref.	Application	Detected Parameter	Sensor Parameters	Interrogation and Detection
Tosi	[74]	RF thermal ablation monitoring	Temperature profile in tissue	CFBG, L = 15 mm, FWHM = 33 nm	Analysis of CFBG spectral regions
Saccomandi	[38]	Laser ablation monitoring	Temperature profile in tissue	CFBG, L = 15 mm, FWHM = 10 nm	Detection of central wavel. and FWHM
Korganbayev	[35]	Laser ablation thermal profiling	Temperature profile in tissue	CFBG, L = 50 mm, FWHM = 40 nm	Spectral reconstruction, white light setup
Palumbo	[36]	RF bipolar resection monitoring	Temperature profile in tissue	CFBG, L = 45 mm, FWHM = 56 nm	Spectral reconstruction, scan. laser setup
Nand	[40]	Positioning of heat source	Temperature hot-spot location	CFBG, L = 15 mm, FWHM = 28 nm	CFBG spectra analysis via FFT
Bettini	[57]	Structural health monitoring	3-point strain gradient	CFBG, L = 30 mm, FWHM = 45 nm	Spectral reconstruction, white light setup
Yashiro	[41]	Monitoring CFRP damage	Multi-point strain peaks	CFBG, L = 30 mm, FWHM ≈ 8 nm	Spectral detection, analysis of strain discontinuities
Yandy	[75]	Detect CFRP defect position	Strain in defect points	CFBG, FWHM ≈ 1 nm	Spectral detection and group delay analysis
Takeda	[76]	Delamination grown in CFRP	Strain discontinuities	CFBG, L = 50 mm, FWHM ≈ 5 nm	Spectral detection, analysis of strain pattern
Wei	[34]	Measure velocity of detonation	Velocity of elongation of CFBG	CFBG, L ≈ 40 mm, FWHM ≈ 30 nm	Dual CFBG, measure CFBG length.
Rodriguez	[77]	Detonation and shock wave propag.	Time response of CFBG elongation	CFBG, L = 10–200 mm, 0.35−3.45 nm/mm	High-speed photodetection
Wydra	[78]	Transmission line sag monitoring	Elongation of CFBG	CFBG, L = 1.7 mm, ψ = 0.1 nm/mm	Detection of spectral shift and FWHM
Chang	[53]	2-dimensional inclinometer	Refractive index discontinuities	Etched CFBGs, Diameter 12 μm, L = 7 mm	2 etched CFBGs, mounted on xy tilt system
Chang	[79]	2-dimensional inclinometer	Refractive index discontinuities	Etched CFBGs, L = 20 mm	2 etched CFBGs, mounted on xy tilt system
Chang	[80]	Liquid-level vertical indicator	Refractive index discontinuity	Etched CFBG, Diameter 12 μm, FWHM = 9.3 nm	White light setup, CFBG in liquid
Osuch	[81]	Temperature independent inclinometer	Tilt angle and temperature	Dual-taper CFBG, L = 20 mm, ψ = 0.135 nm/mm	White light setup, spectral analysis
Swart	[81]	Pressure and position sensing	Pressure and position	CFBG, L = 100 mm, FWHM = 1.1 nm	Mach-Zehnder setup, RF detector
Wang	[82]	Strain measurement	Distributed strain	CFBGs, L = 17 mm	2-CFBG system, linear chirp interrogation
Liu	[83]	Strain and temperature measurement	Distributed strain and temperature	CFBG, L ≈ 115 mm, Birefr. fiber	Mach-Zehnder Interferometer
Osuch	[84]	Strain measurement	Strain/force	Dual-taper CFBG, L = 20 mm, Two-sided taper	White light setup, spectral analysis
Sun	[84]	Refractive index measurement	Refractive index change	LPG/CFBG, L ≈ 20 mm, FWHM = 16 nm	Cladding mode analysis LPG-induced
Duraibabu	[85]	Dual temper. profile + pressure	Temperature profile, pressure	EFPI/CFBG, FWHM ≈ 3 nm	White light setup, dual sensor
